# Mesopancreas—Anatomical Insights and Its Implications for Diagnosis and Clinical and Surgical Practice

**DOI:** 10.3390/diagnostics15070914

**Published:** 2025-04-02

**Authors:** Florin-Mihail Filipoiu, Georgian-Theodor Badea, Mihaly Enyedi, Ștefan Oprea, Zoran-Florin Filipoiu, Daniela-Elena Gheoca Mutu

**Affiliations:** 1 Discipline of Anatomy, Department 2–Morphological Sciences, “Carol Davila” University of Medicine and Pharmacy, 020021 Bucharest, Romania; florin.filipoiu@umfcd.ro (F.-M.F.); mihaly.enyedi@umfcd.ro (M.E.); stefan.oprea@umfcd.ro (Ș.O.); daniela-elena.mutu@umfcd.ro (D.-E.G.M.); 2 Doctoral School, “Carol Davila” University of Medicine and Pharmacy, 020021 Bucharest, Romania; 3 Faculty of Medicine, “Carol Davila” University of Medicine and Pharmacy, 020021 Bucharest, Romania; zoran-florin.filipoiu2022@stud.umfcd.ro

**Keywords:** mesopancreas, retropancreatic lamina, celiac nervous ganglia, pancreatic head cancer, anatomy, pancreas, dissection, embriology, TMpE, pancreatic surgery

## Abstract

**Background:** The concept of mesopancreas is frequently discussed in the surgical literature as the neural pathway for metastatic spread in pancreatic head cancer. It generally refers to a retro-pancreatic plane that should be resected to reduce the incidence of regional metastases. However, this concept remains poorly defined, both embryologically and anatomically. Our objective was to establish a clear embryological and anatomical definition of the mesopancreas, making anatomical data more applicable in surgical practice. **Methods:** We examined seven cadavers (5 males, 2 females, aged 62–71) with no medical or surgical history, preserved in 9% formalin at Carol Davila University’s Anatomy Department. Regional dissections were performed in successive planes, highlighting the celiac ganglia and the associated network of neural connections that comprise the mesopancreas. **Results:** Our study defines the “mesopancreas” as remnants of primordial mesenteries that coalesced into the Treitz fascia. We identified its functional components as nerve fibers linking the celiac ganglia and superior mesenteric plexus to the pancreas, along with vascular structures, lymphatics, and connective and adipose tissue. These components likely contribute to regional metastasis in pancreatic head cancer. While resection of the mesopancreas could help prevent metastasis, its complex anatomy and proximity to major vessels pose significant surgical challenges. **Conclusions:** Based on our findings, we propose a plausible definition for the term “mesopancreas”. It encompasses the structures that originated as part of the primordial mesenteries, which subsequently coalesced, resulting in the formation of the Treitz fascia. In essence, the mesopancreas is the functional content of a former mesentery.

## 1. Introduction

The term “mesopancreas” is widely used in the surgical literature to describe the neural pathway implicated in the metastatic spread of pancreatic head cancer. It has emerged as a focal point in pancreatic surgery, particularly concerning pancreaticoduodenectomy (PD) for malignancies of the pancreatic head [1], and its significance lies in its role as a conduit for tumor infiltration and metastasis, making it a critical area for achieving negative resection margins during PD [2]. Generally speaking, the mesopancreas is associated with a retro-pancreatic plane that should be resected to minimize the occurrence of regional metastases. First introduced in 2007 by Gockel and colleagues, the mesopancreas was described as a connective-neurovascular sheet located retroportally, posterior to the pancreatic head. According to Gockel, the mesopancreas is a well-organized structure extending from the posterior surface of the pancreatic head, retroportally, to the level of the superior mesenteric artery. Their study also emphasized the critical role of mesopancreatic lymphatic structures in facilitating metastatic spread [3]. Perineural tumor metastasis was observed in up to 77% of resection specimens from patients with pancreatic head carcinoma [4,5]. Such perspectives support the notion that complete resection of the mesopancreas during PD could potentially improve oncological outcomes by reducing local recurrence rates.

However, certain studies have challenged the existence of the mesopancreas as a distinct anatomical entity, leading to ongoing controversies [6] and debates regarding its precise anatomical boundaries and the extent of involvement in disease progression. Some researchers viewed it as an artifact of dissection, lacking distinct anatomical boundaries and questioning its classification as a true mesentery. They argued that the absence of a fibrous capsule or fascia, similar to the mesorectum, makes it invisible and difficult to identify during surgery [6]. This divergence of opinion underscores the need for a standardized definition and a clearer understanding of the mesopancreas’s anatomy and its relevance in pancreatic surgery [1]. Further research is vital to delineate this structure precisely and develop surgical approaches that ensure its complete excision, potentially improving outcomes for patients undergoing duodenopancreatectomy for pancreatic head tumors.

From a localization perspective, the existing literature provides inconsistent descriptions. Nonetheless, it is generally accepted that the mesopancreas refers to a retro-pancreatic neural plane containing nerves, blood vessels, and lymphatic vessels, with an anatomical relationship to the right side of the superior mesenteric artery [7,8,9].

From a developmental standpoint, the pancreas’s evolution is closely tied to the fusion of the dorsal and ventral mesogastrium and the development of pancreatic buds. The pancreas originates from two embryonic buds: the dorsal pancreatic bud, which arises from the dorsal mesogastrium, and the ventral pancreatic bud, which develops from the ventral mesogastrium. The dorsal bud contributes to the formation of the body, isthmus, and tail of the pancreas, while the ventral bud, initially associated with the hepatic bud, forms the pancreatic head and uncinate process [10]. During duodenal rotation, the ventral bud migrates dorsally and eventually fuses with the dorsal bud, occurring in the 6th to 7th week of embryonic development. Through duodenopancreatic coalescence, the dorsal and ventral mesogastrium disappear, leaving the pancreas as a secondary retroperitoneal organ. The remaining structures from the primordial mesenteries—such as blood vessels, nerves, and lymphatic tissue—become integrated into the mesopancreatic plane, which assumes a significant functional role [11].

This review aims to elucidate the anatomical and embryological foundations of the mesopancreas, explore its clinical implications in pancreatic surgery, and discuss current controversies and advancements in its management. By synthesizing data from the existing literature, we want to provide a comprehensive understanding of the mesopancreas and its impact on surgical strategies and patient outcomes. Building on these foundational concepts, we aim to present the information in a structured and logical manner.

## 2. Materials and Methods

The dissection study was conducted on seven cadavers, aged between 62 and 71 years (five males and two females), with no prior medical or surgical history. The cadavers were preserved using a 9% formaldehyde solution in the dissection laboratory of the Department of Morphology at the Carol Davila University of Medicine and Pharmacy in Bucharest. The use of cadavers was carried out following the Cadaver Handling Law [12], which regulates the use of cadavers in anatomy laboratories in Romania.

The dissections were performed in successive planes to replicate the surgical steps closely. The results of these meticulous dissections were photographed, with particular emphasis on the regions surrounding the mesopancreas. All images were digitally edited without altering the scientific content.

To enhance the understanding of the mesopancreatic region from both anatomical and radiological perspectives, we attempted to establish a correlation between the anatomical aspects identified during dissection and their radiological counterparts through the analysis of computed tomography (CT) scans. The radiological images are anonymized images from the Database of the Morphology Department.

## 3. Results

### 3.1. Pancreas Development

To summarize, the duodenum initially appears as a loop located in the sagittal plane, with its convexity directed anteriorly. It is anchored anteriorly by the ventral mesentery (the lower part of the ventral mesogastrium). At the same time, posteriorly, the loop is attached to the posterior trunk wall via a dorsal mesentery (primitive dorsal mesentery or dorsal mesogastrium). Within the ventral mesentery, the ventral pancreatic bud (more precisely, the hepatobiliopancreatic bud) develops from the duodenal wall, while the dorsal pancreatic bud develops in the dorsal mesentery at a level superior to the ventral one. Through the rotation of the ventral pancreatic bud, it, along with its mesentery, changes position from ventral to dorsal [13].

**Figure 1:**
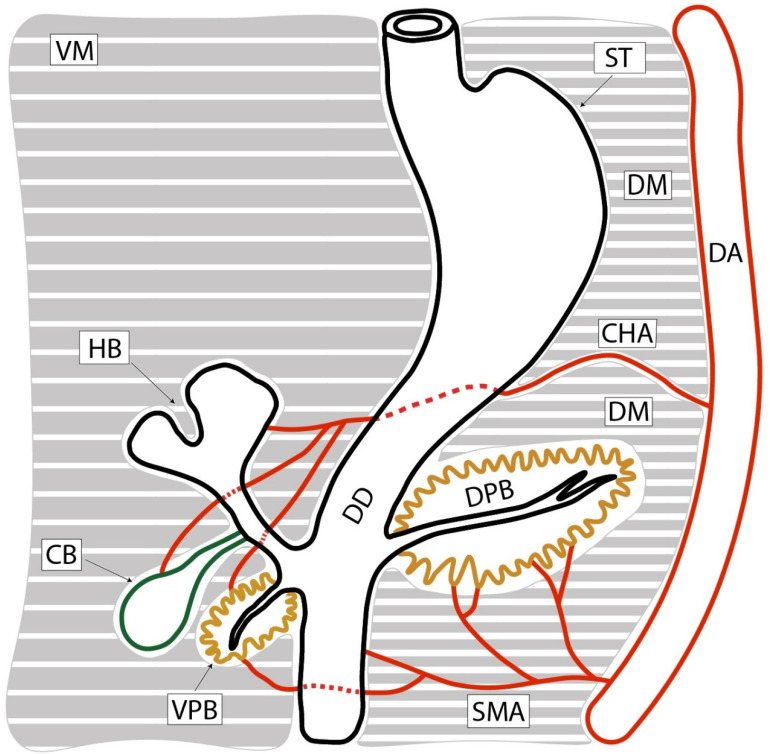
Vascularization sources of the pancreatic primordia, considering the final adult configuration. The pancreas is vascularized by the celiac trunk and the superior mesenteric artery. Abbreviations: SMA—superior mesenteric artery; VPB—ventral pancreatic bud; CB—cystic bud; HB—hepatic bud; DD—duodenum; DPB—dorsal pancreatic bud; CHA—common hepatic artery; DA—dorsal aorta; ST—stomach; VM—ventral mesogastrium; DM—dorsal mesogastrium.

Each of the two mesenteries has its own arterial supply: the ventral mesentery is supplied by the proper hepatic artery (which serves all derivatives of the bud), while the superior mesenteric artery supplies the dorsal mesentery. Both mesenteries have lymphatic drainage directed to lymph nodes associated with the origins of the major arteries: the celiac trunk and, respectively, the superior mesenteric artery (Figure 1).

Autonomic innervation reaches the pancreas via both mesenteries and originates from the celiac ganglia and the superior mesenteric ganglion. In other words, the mesentery of the ventral pancreatic bud has its own vascularization and innervation (celiac nerve ganglion, lymph nodes around the celiac trunk, and the proper hepatic artery). The dorsal pancreatic bud is supplied by the superior mesenteric artery, with lymphatic drainage into the mesenteric and celiac lymph nodes, and receives nerve fibers from the celiac ganglion, but predominantly from the superior mesenteric plexus [14].

**Figure 2:**
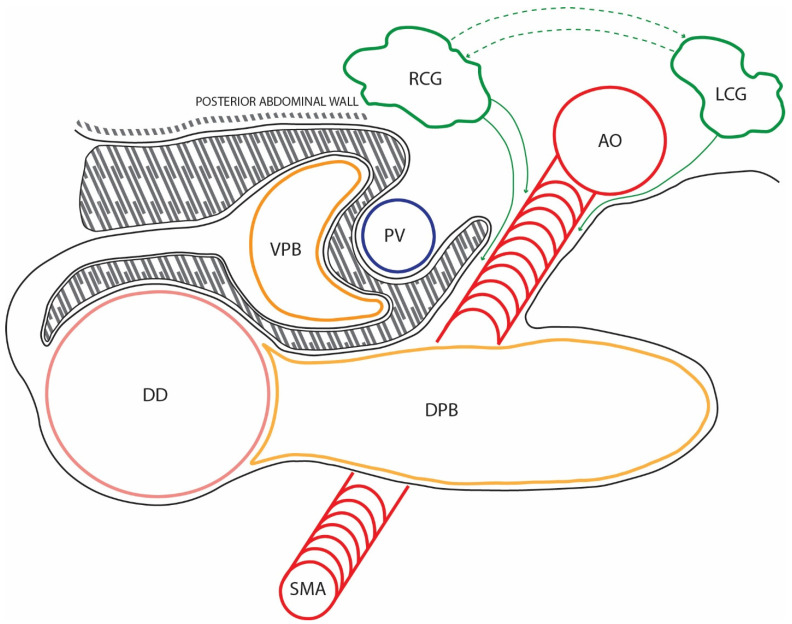
The shaded area represents the loose connective tissue of the Treitz coalescence fascia. It was formed through the fusion of the posterior parietal peritoneum, the mesentery of the dorsal bud, and the mesentery of the ventral bud. As observed, the portal vein (PV) divides the Treitz fascia into a preportal sheet and a retroportal sheet. The solid line represents the peritoneum and the dashed line corresponds to the posterior abdominal wall. Abbreviations: DPB—dorsal pancreatic bud with its mesentery; VPB—ventral pancreatic bud, after rotation, pulling part of the ventral mesogastrium with it; SMA—superior mesenteric artery, surrounded by the superior mesenteric plexus, marking the boundary of the mesopancreatic sheet; RCG—right celiac ganglion (in green), sending efferents (green arrows) to the superior mesenteric plexus and the mesopancreatic sheet; LCG—left celiac ganglion, sending efferents to the superior mesenteric plexus and the right celiac ganglion; DD—duodenum; AO—aorta.

The duodenal loop shifts its position from a sagittal to a horizontal orientation through a rightward displacement caused by the growth and positional changes in the liver. As a result of this positional modification, the mesentery of the posterior pancreatic bud covers the origin of the portal vein and the ventral pancreatic bud along with its mesentery. The connective structures of the mesenteries fuse during a process called coalescence, forming the Treitz retro-duodenopancreatic fascia [15,16]. Consequently, the mesenteries of the pancreatic buds disappear; however, it is essential to note that their contents remain embedded in the Treitz fascia (Figure 2).

Based on these deductions, we can conclude that the mesopancreas is a composite of neural, lymphatic, and connective-adipose structures that initially belonged to the mesenteries of the two pancreatic buds. Essentially, the mesenteries disappeared through coalescence, while their contents remained retro-pancreatic and around the superior mesenteric artery. In this arrangement, the described retro-pancreatic environment justifies its designation as the mesopancreas, representing a structure that was once part of a vanished mesentery.

### 3.2. Mesopancreas—Definition and Demonstration Through Dissection: Dissection of the Celiac Ganglia

**Figure 3:**
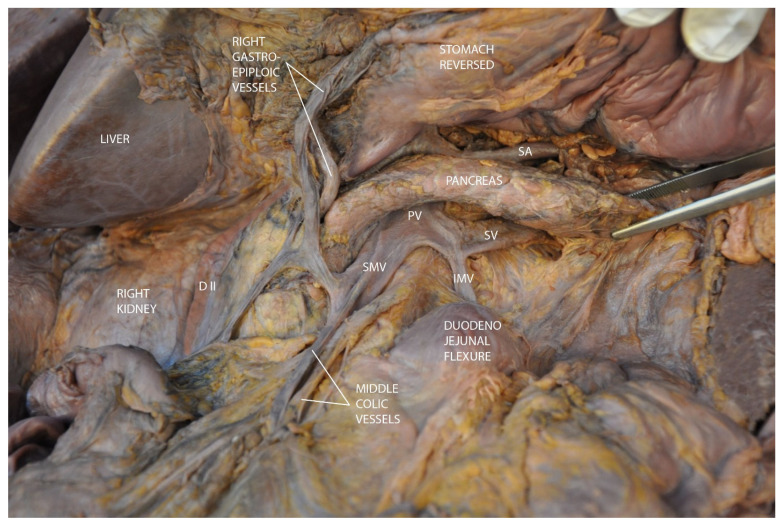
Formation of the portal vein behind the pancreas. Abbreviations: DII—second segment of the duodenum, SMV—superior mesenteric vein, PV—portal vein, SA—splenic artery, SV—splenic vein, IMV—inferior mesenteric vein.

After exposing the pancreas, gentle traction on its lower edge allows for visualization of the portal vein formation in the retropancreatic space (Figure 3).

**Figure 4:**
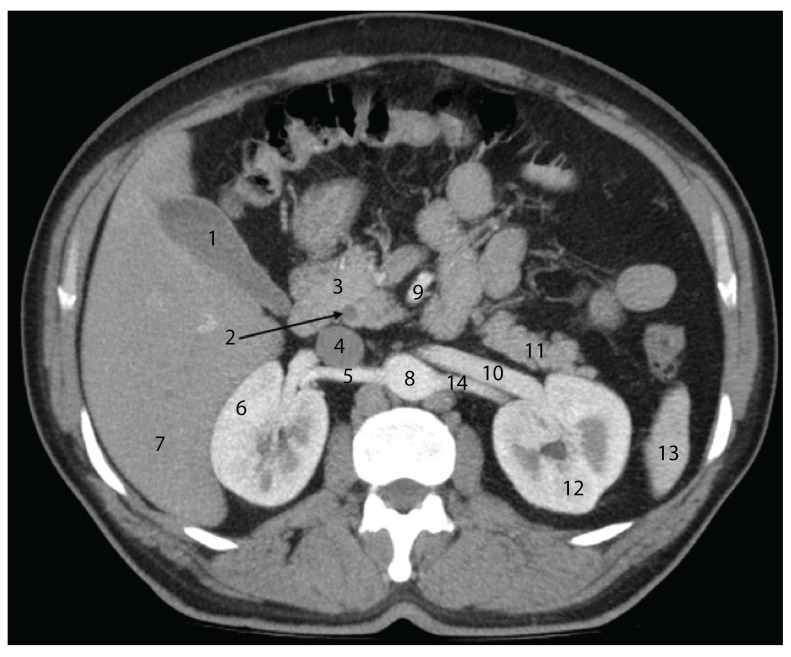
Axial CT section in the arterial phase, at the level of the L1 vertebral body, illustrating the anatomical relationships of the cephalo-corporeal region of the pancreas. 1—Gallbladder; 2—common bile duct; 3—head of the pancreas; 4—inferior vena cava; 5—right renal artery; 6—right kidney; 7—right hepatic lobe; 8—abdominal aorta; 9—superior mesenteric artery; 10—left renal vein; 11—tail of the pancreas; 12—left kidney; 13—spleen; 14—left renal artery.

In the axial section shown in Figure 4, the pancreatic head is easily identifiable, having a close posterior relation to the common bile duct. Posterior to this, the inferior vena cava can be observed. Notably, the portal vein is not visible in this section; it appears in a slightly more cranial slice.

The relationships between the pancreas and the renal vessels are also evident, with the renal veins occupying the anterior plane of the renal pedicle. Contrary to classical anatomical descriptions, the tail of the pancreas does not reach the splenic hilum in this section. During dissection, we observed a transverse curvature of the pancreatic body, which explains why the entire pancreas may not be captured within a single axial plane.

**Figure 5:**
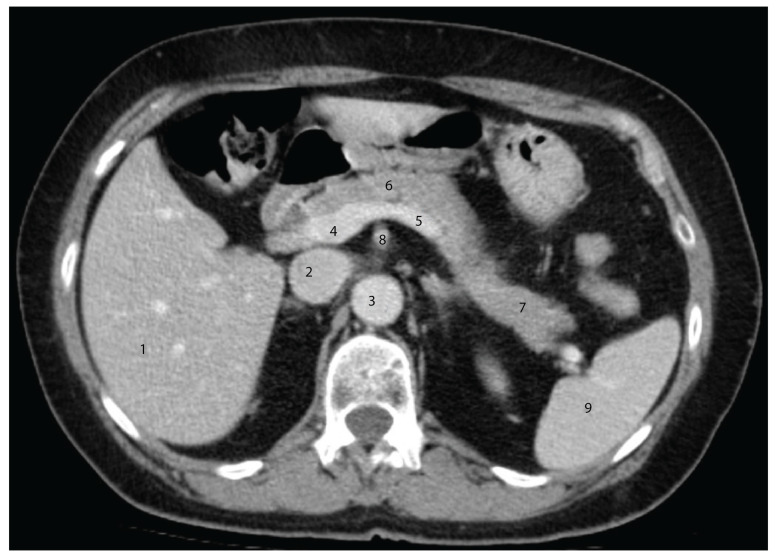
Axial CT section in the venous phase at the inferior border of the T12 vertebral body. 1—Right hepatic lobe; 2—inferior vena cava; 3—descending abdominal aorta; 4—portal vein; 5—splenic vein; 6—body of the pancreas; 7—tail of the pancreas; 8—superior mesenteric artery; 9—spleen.

In Figure 5, the body and tail of the pancreas are visible, without the pancreatic head. The portal vein is clearly seen, along with the confluence of the splenic vein, which is visible along its distal course. Posterior to the portal vein, the inferior vena cava can be observed.

These observations highlight the importance of understanding the anatomical relationship between the portal vein and the inferior vena cava at their crossing point. The image is located just below the level of the emergence of the superior mesenteric artery, the inferior wall of which can be seen with relative difficulty. The tail of the pancreas reaches the splenic hilum.

**Figure 6:**
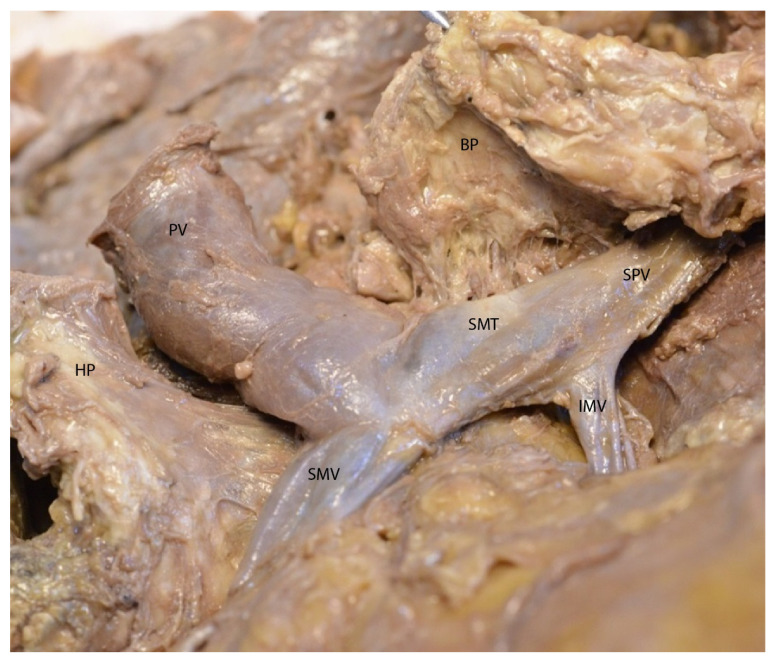
Transection of the pancreatic isthmus. Visualization of the portal vein. Abbreviations: HP—head of the pancreas (retracted), BP—body of the pancreas (retracted), SMV—superior mesenteric vein, SMT—splenomesenteric trunk, IMV—inferior mesenteric vein, SPV—splenic vein, PV—portal vein.

In Figure 6, we performed a transverse section of the pancreas, separating the head from the body. The classical formation of the portal vein is visible in the image plane.

**Figure 7:**
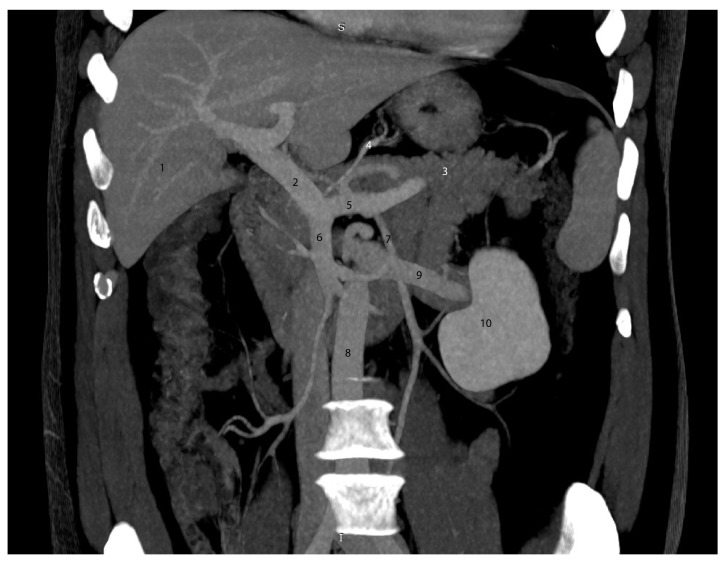
Mesenteric vein confluence—CT MPR reconstruction in the coronal plane during the venous phase, with 26 mm MIP (maximum intensity projection). 1—Liver; 2—portal vein; 3—pancreas; 4—left gastric vein vein; 5—splenic vein; 6 – superior mesenteric vein; 7 – inferior mesenteric vein; 8 – inferior vena cava; 9 – left renal vein; 10 – left kidney.

In Figure 7, we highlight the portal vein formation. In this image, the left gastric vein drains into the splenoportal confluence. We have placed the CT image (Figure 7) alongside the dissection image as one of the most illustrative examples of the mutual usefulness of anatomical and radiological correlation in identifying key structures. We believe that this approach represents the most effective methodology for the radiological interpretation of anatomical data.

**Figure 8:**
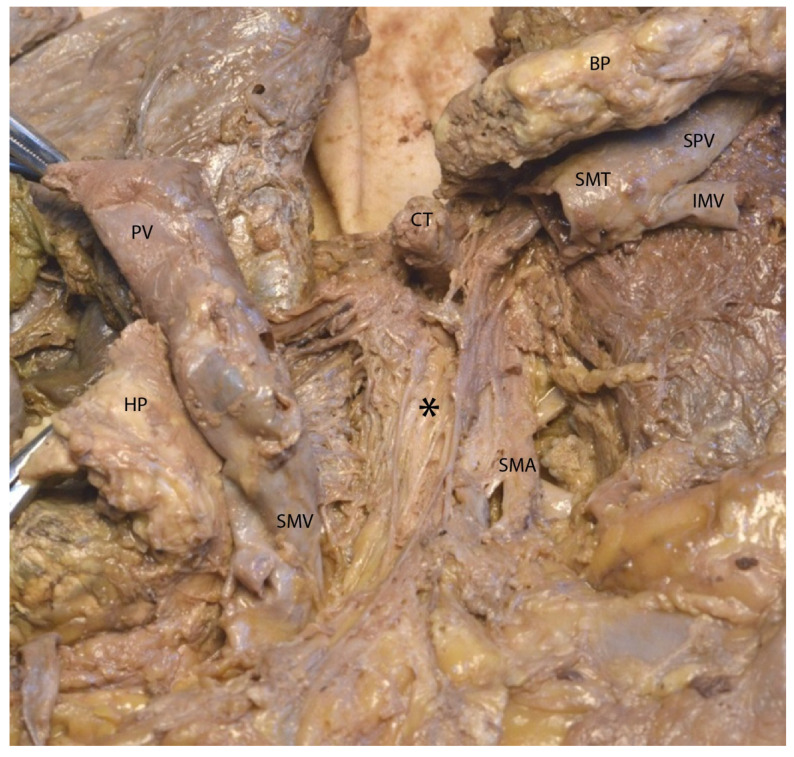
Transection of the splenomesenteric trunk on the left side of the portal vein to expose the mesopancreatic plane. Abbreviations: HP—head of the pancreas (retracted), BP—body of the pancreas (retracted), SMV—superior mesenteric vein, SMT—spleno-mesenteric trunk, IMV—inferior mesenteric vein, SPV—splenic vein, PV—portal vein, SMA—superior mesenteric artery, CT—celiac trunk, * (asterisk)—mesopancreas.

**Figure 9:**
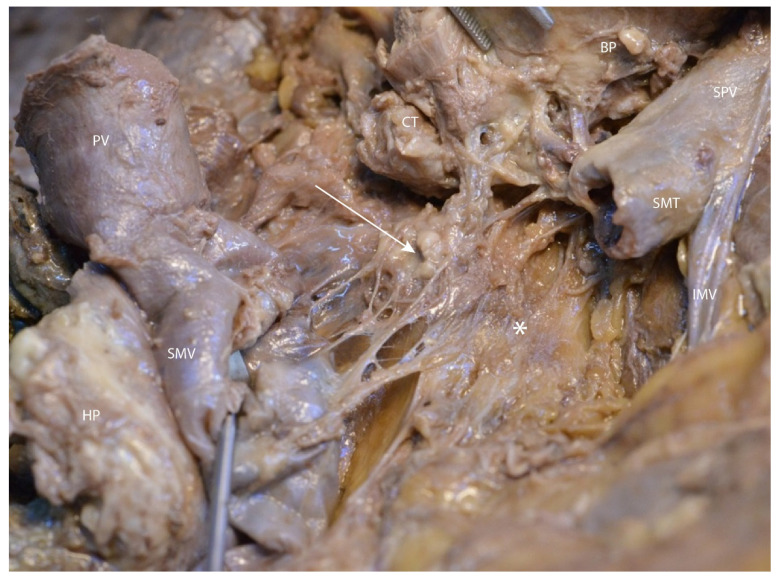
Visualization of retrolaminar lymph nodes. Abbreviations: PV—portal vein, SMV—superior mesenteric vein, HP—head of the pancreas (retracted), CT—celiac trunk, BP—body of the pancreas (retracted), SMT—splenomesenteric trunk, IMV—inferior mesenteric vein, SPV—splenic vein, * (asterisk)—mesopancreas, arrow—retrolaminar lymph nodes.

In the following figures (Figure 8 and Figure 9), after transecting the portal vein at the level of the splenomesenteric trunk and retracting the transected structures to the right and left, we exposed the mesopancreatic plane (retroportal) and a group of small retroportal lymph nodes, predominantly located posterior to this plane.

**Figure 10:**
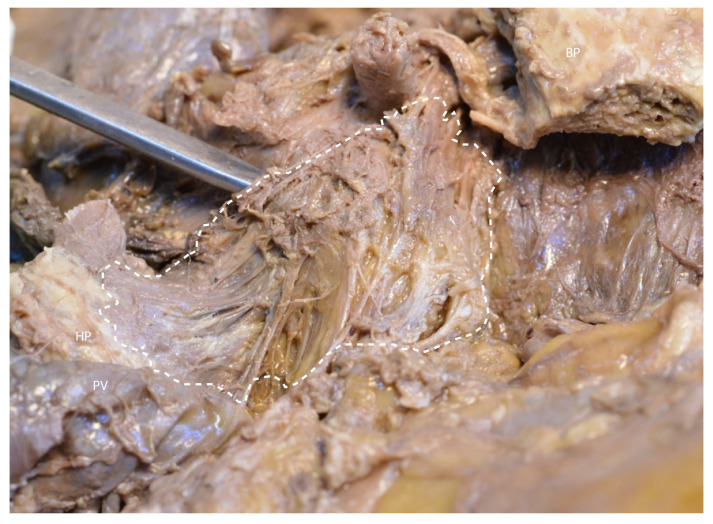
Visualization of the retroportal mesopancreatic plane. Abbreviations: PV—portal vein, HP—head of the pancreas (retracted), BP—body of the pancreas (retracted), dash-circled structure—mesopancreas.

In Figure 10, the retroportal mesopancreatic plane is held with forceps for clear demonstration.

**Figure 11:**
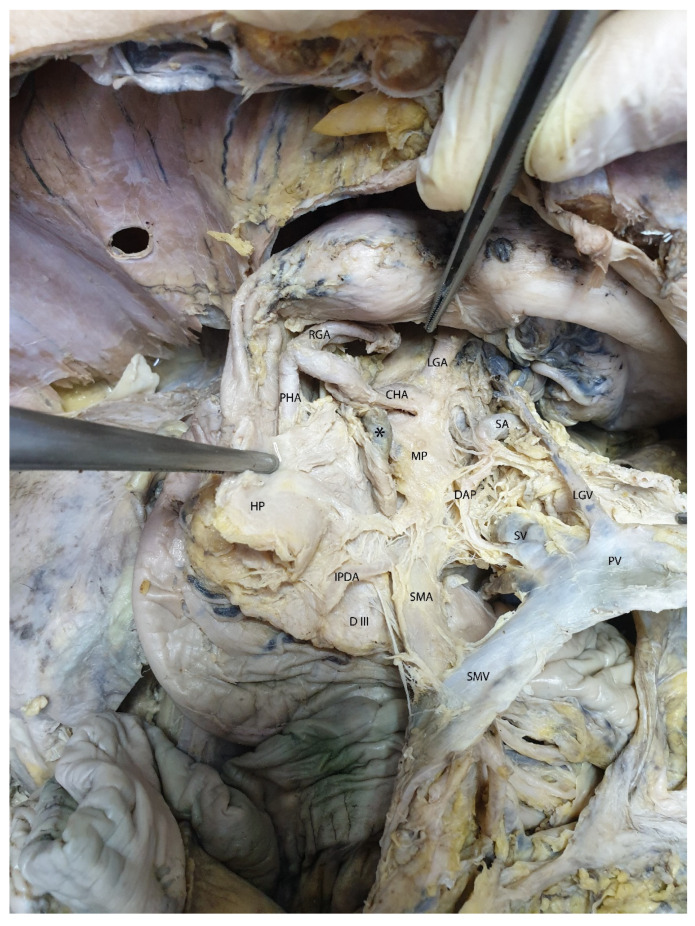
The mesopancreatic plane and a retro-pancreatic lymph node, located anterior and to the right of the superior mesenteric artery. Abbreviations: HP—head of the pancreas (retracted), PHA—proper hepatic artery, RGA—right gastric artery, CHA—common hepatic artery, LGA—left gastric artery, SA—splenic artery, LGV—left gastric vein, SV—splenic vein, PV—portal vein, SMV—superior mesenteric vein, SMA—superior mesenteric artery, MP—mesopancreas, D III—third segment of the duodenum, IPDA—inferior pancreatico-duodenal artery, DAP—dorsal artery of the pancreas, * (asterisk)—prelaminar lymph node.

In Figure 11, after the transection of the pancreas and the portal vein, the mesopancreatic plane and a retro-pancreatic lymph node are clearly visible. Following the retraction of the portal vein and the transected pancreatic segments, the retro-pancreatic plane is distinctly observed, located anterior and to the right of the superior mesenteric artery. The presence of the inferior pancreaticoduodenal artery and the dorsal pancreatic artery is also noted.

**Figure 12:**
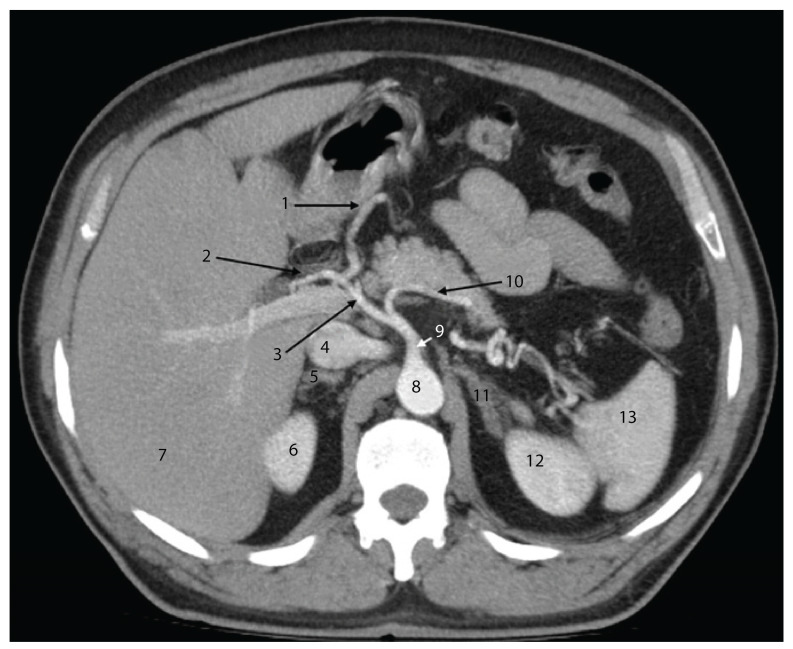
Visualization of the celiac trunk in an axial CT section during the arterial phase (MIP reconstruction with 4 mm slice thickness). 1—Right gastric artery; 2—proper hepatic artery; 3—common hepatic artery; 4—inferior vena cava; 5—right adrenal gland; 6—superior pole of the right kidney; 7—right hepatic lobe; 8—abdominal aorta; 9—celiac trunk; 10—splenic artery; 11—left adrenal gland; 12—superior pole of the left kidney; 13—spleen.

To support a three-dimensional understanding of the mesopancreatic region and its essential vascular relationships, a CT image illustrating the origin of the celiac trunk and its main branches in relation to the pancreas was included (Figure 12). This area is of major importance in defining the mesopancreas, as the celiac trunk and its associated plexus represent the origin of the nerve fibers that traverse the mesopancreatic region.

Furthermore, the relationship between the splenic artery, with its tortuous course, and the superior surface of the pancreas provides a valuable radiological landmark for delineating the upper boundary of the mesopancreas. Additionally, the identification of the adrenal glands and the diaphragmatic crura allows for accurate anatomical orientation in both the axial and coronal planes, facilitating correlation with the findings obtained through dissection.

**Figure 13:**
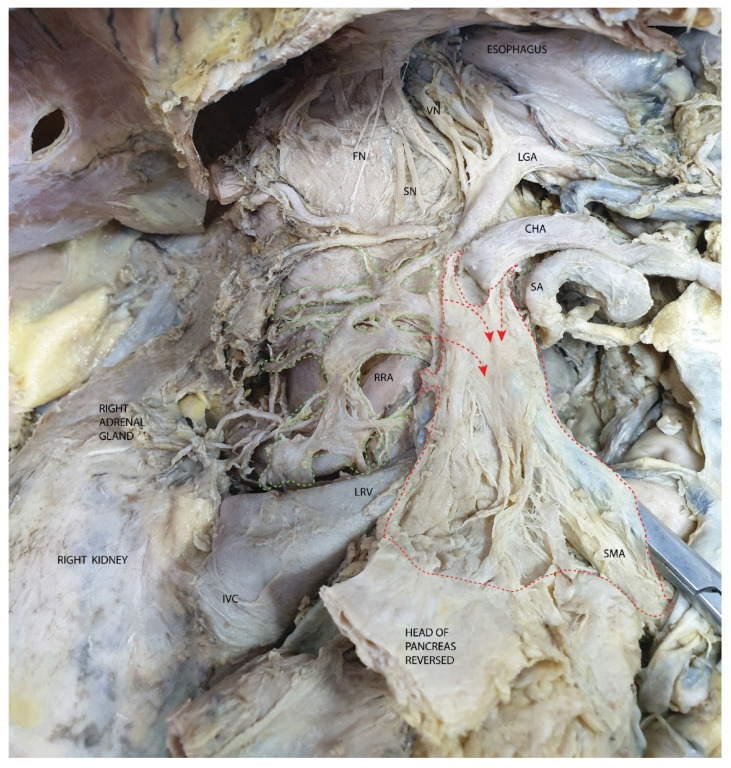
The right celiac ganglion and its neural connections to the right renal ganglion, sympathetic chain, and posterior vagal trunk. Abbreviations: FN—phrenic nerve, SN—sympathetic nerves, VN—vagus nerve, LGA—left gastric artery, CHA—common hepatic artery, RRA—right renal artery, IVC—inferior vena cava, LRV—left renal vein, SMA—superior mesenteric artery, red arrows—afferent fibers from the celiac ganglion to the mesopancreas, highlighted with green dots—right renal and celiac ganglia, highlighted with red dots—mesopancreas. In the figure, the clamp is inserted in the cleavage plane between the superior mesenteric artery and the mesopancreas.

In Figure 13, we have the best demonstration of the neural component of the mesopancreas, showing the origin of the nerve fibers. Highlighted in green is the right celiac ganglion in relation to the right renal ganglion. From the celiac ganglion, both efferent fibers to the mesopancreatic plane and transverse fibers connecting to the left celiac ganglion can be observed. For orientation, the branches of the celiac trunk are indicated. In the upper part of the image, the neural connections of the celiac ganglion with the phrenic nerve, the sympathetic chain, and the posterior vagal trunk are visible.

**Figure 14:**
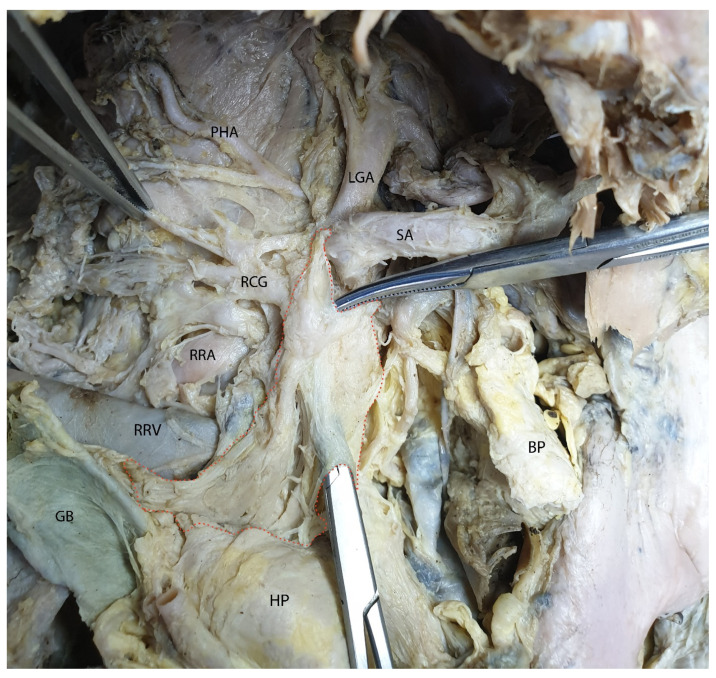
Mesopancreas loaded on two surgical clamps. Abbreviations: RRV—right renal vein; RRA—right renal artery; GB—gallbladder; RCG—right celiac ganglion; PHA—phrenic artery; SA—splenic artery; LGA—left gastric artery; BP—body of the pancreas; HP—head of the pancreas; inside the red dotted circled area—the mesopancreas.

In the following figure (Figure 14), the mesopancreas was loaded onto two surgical clamps, which were inserted between the mesopancreatic blade and the superior mesenteric artery.

**Figure 15:**
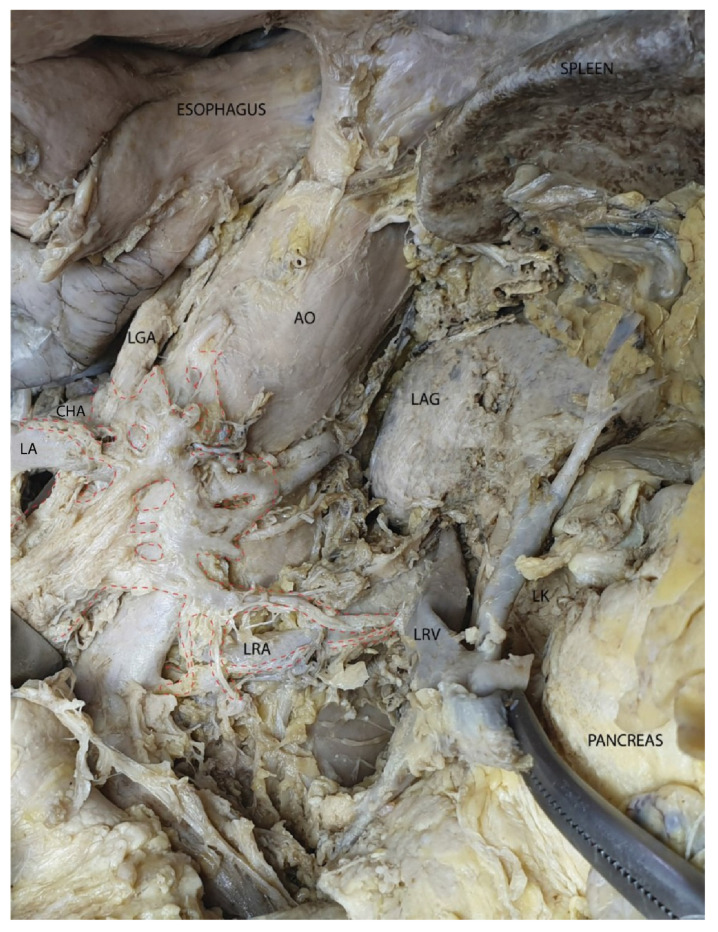
The left celiac ganglion and its efferent connections to the mesopancreas, the right celiac ganglion, and the left renal ganglion (circled by the red dotted line). Abbreviations: AO—abdominal aorta, LGA—left gastric artery, CHA—common hepatic artery, LA—lienal artery (retracted), LAG—left adrenal gland, LRA—left renal artery, LRV—left renal vein, LK—left kidney.

In Figure 15 the left celiac ganglion is located in relation to the left side of the aorta, between it and the left renal artery. Its efferent fibers take three main directions: toward the mesopancreas, the right celiac ganglion, and the left renal ganglion. Notably, the origin of the celiac trunk is practically surrounded by neural connections between the two celiac ganglia.

**Figure 16:**
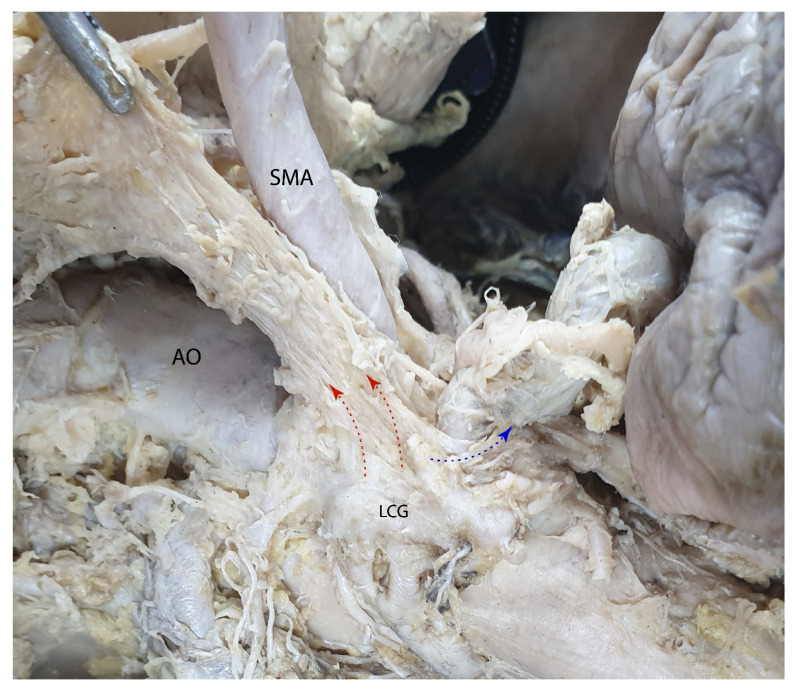
The superior mesenteric plexus is located within the periarterial tissue surrounding the superior mesenteric artery. Abbreviations: AO—aorta; LCG—left celiac ganglion; SMA—superior mesenteric artery; red arrows—efferent fibers from the left celiac ganglion to the superior mesenteric plexus; blue arrow—nervous fibers from the left celiac ganglion to the right celiac ganglion.

From both celiac ganglia, nerve fibers emerge to form the superior mesenteric nerve plexus around the superior mesenteric artery. This plexus provides sympathetic innervation to all abdominal organs supplied by the superior mesenteric artery. In the following figure, the origin of the SMA is shown from the left side to highlight the perimesenteric efferents of the left celiac ganglion (Figure 16).

**Figure 17:**
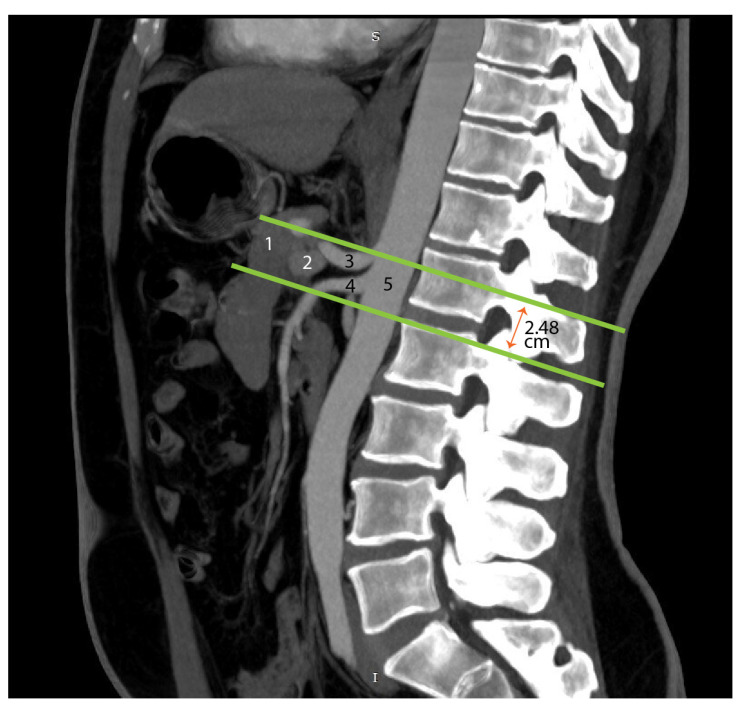
Computed tomography depiction of the mesopancreatic region. The green lines outline the boundary of the mesopancreas, a region identified as the retro-pancreatic retro-portal tissue. In the figure, the inferior boundary of the mesopancreas is 2.48 cm below the origin of the celiac trunk. 1—pancreas; 2—portal vein; 3—celiac trunk 4—superior mesenteric artery; 5—abdominal aorta.

The radiological evaluation of retroperitoneal structures remains a true challenge for young specialists in radiology and medical imaging. The deep location of retroperitoneal organs, variability in vascular pathways, the morphology and size of the pancreas, as well as the presence of structures that are difficult to visualize, all contribute to the complexity of detailed imaging diagnosis in this region. However, some authors describe the superior, inferior, anterior, and posterior resection margins of TMpE as forming an inverted triangular region, referred to as the mesopancreas triangle [9]. According to them, this triangle is bounded by a base resting on the posterior surface of the superior mesenteric vein (SMV) and portal vein (PV), and a summit located on the anterior surface of the aorta, between the origins of the celiac trunk and the superior mesenteric artery (SMA). We assessed the position of the mesopancreatic space in relation to the vertebral column, the cranio-caudal diameter of the pancreas in sagittal sections, the distance between the posterior margin of the pancreas and the aorta. Measurements were performed in a sagittal plane that captured the origin of the superior mesenteric artery and celiac trunk from the descending abdominal aorta (Figure 17).

## 4. Discussion

From a semantic perspective, the prefix “meso” in the term “mesopancreas” can be misleading when defining the structure. At first glance, it might suggest the presence of a mesentery associated with the pancreas. However, it is well established that the pancreas is a secondary retroperitoneal organ that lacks a mesentery. During intrauterine development, the pancreatic primordia, represented by the dorsal and ventral buds, possessed a well-defined mesentery as part of the dorsal and ventral mesogastrium. However, these mesenteric structures disappear during the process of duodenopancreatic coalescence, resulting in the pancreas lacking a mesentery in its mature form.

The use of the prefix “meso” is justified, however, to denote the structures that were initially located between the two layers of the mesenteries during embryonic development and remain functional. These structures are not only significant in understanding the anatomy and physiology of the pancreas but also critical in the context of surgical intervention, where the deep location of the mesopancreatic plane and its proximity to major vascular and nervous structures present considerable challenges and risks.

### 4.1. Surgical Complications

The primary component of the mesopancreatic plane comprises the afferent and efferent fibers of the left and right celiac ganglia. Both celiac ganglia contribute to the formation of the mesopancreatic plane and the superior mesenteric plexus. A defining feature of the mesopancreatic plane is its anatomical relationship with the superior mesenteric artery and its proximity to the portal vein. Therefore, vascular injuries during mesopancreatic dissection can lead to life-threatening hemorrhage. Furthermore, chronic inflammatory conditions, such as pancreatitis, can exacerbate this risk by causing fibrosis and obscuring anatomical landmarks, thereby complicating dissection and increasing the likelihood of vascular injury. Fibrotic changes in the mesopancreas may also impair the ability to achieve clear resection margins, affecting oncological outcomes [17]. Inadequate understanding of the vascular anatomy or technical errors during dissection may result in uncontrollable bleeding, increasing morbidity and mortality rates.

The celiac ganglia, part of the prevertebral sympathetic ganglia, are deep neural structures that regulate the sympathetic activity of multiple abdominal viscera. Disruption of interganglionic connections within this region can impair the sympathetic regulation of these organs. For instance, injury to the superior mesenteric plexus can result in sympathetic denervation of the intestinal loops and the right colon. Some of the most frequent complications reported were delayed gastric emptying, diarrhea, and pancreatic exocrine dysfunction [17]. Chronic pancreatitis may also contribute to neuropathic changes within the mesopancreas, further complicating its dissection. A systematic review of postoperative complications following PD revealed that surgical expertise and preoperative planning were critical in minimizing these risks [17]. Studies also recommend preoperative imaging to delineate the vascular and neural anatomy of the mesopancreas, thus reducing intraoperative errors [18]. Furthermore, advancements in robotic and laparoscopic techniques have shown promise in improving surgical precision and minimizing complications [19,20].

### 4.2. Lymphatic Spread

An important aspect that needs to be emphasized is the presence of lymph nodes situated both superficially and deeply relative to the mesopancreatic plane. By having a dense network of lymphatic vessels and nodes, this anatomical region plays a vital role in the metastatic spread of pancreatic cancer [21]. Tumor cells often disseminate through these lymphatic pathways, making the mesopancreas a critical area for lymph node dissection during pancreatic ductal adenocarcinoma (PD). The involvement of mesopancreatic lymph nodes has been strongly correlated with advanced disease staging and a poorer prognosis [22]. Emerging techniques, such as sentinel lymph node mapping, are being explored to enhance the precision of lymphatic dissection in the mesopancreatic region [23]. These advancements are particularly relevant in stratifying patients based on their nodal involvement and tailoring adjuvant therapy accordingly. However, lymphatic mapping studies have demonstrated that the lymphatic pathways of the mesopancreas frequently harbor micrometastases, which may remain undetected in preoperative imaging but significantly influence prognosis [24]. The presence of these micrometastases in resected mesopancreatic specimens has underscored the necessity of extensive lymphadenectomy to ensure higher oncological clearance.

### 4.3. Oncological Outcomes

The mesopancreas plays a pivotal role in the spread of pancreatic tumors, making the status of mesopancreatic margins a critical factor in pancreaticoduodenectomy (PD). Studies have demonstrated that patients with positive mesopancreatic margins exhibit higher rates of local recurrence and shorter disease-free survival compared to those with negative margins [25]. However, the long-term impact of mesopancreatic margin status on overall survival remains inadequately understood due to variability in surgical techniques and pathological assessments. Further research is needed to correlate mesopancreatic margin status with survival outcomes, taking into account the role of adjuvant therapies and the extent of lymphatic and neural invasion. Multicenter trials and standardized histopathological criteria for margin assessment will be key in addressing this gap, ultimately guiding surgeons in optimizing oncological clearance during PD [26].

### 4.4. Comparison with the Mesorectum

In rectal cancer surgery, the concept of the mesorectum has been pivotal in enhancing surgical outcomes. The introduction of total mesorectal excision (TME) by Heald et al. in 1982 revolutionized rectal cancer treatment by emphasizing the en bloc removal of the rectum along with its mesorectal envelope [27], leading to significant reductions in local recurrence rates and improvements in survival [28]. Drawing parallels from this approach, the mesopancreas has been proposed as an oncologic unit in pancreatic surgery, particularly during pancreaticoduodenectomy (PD) for pancreatic head malignancies.

The concept of total mesopancreas excision (TMpE) aims to achieve negative resection margins by removing the mesopancreas en bloc with the pancreatic head [29]. This approach is analogous to TME in rectal cancer surgery, where the mesorectum is excised intact to minimize local recurrence. Studies have demonstrated that TMpE can increase R0 resection rates and reduce locoregional recurrence, thereby improving oncological outcomes. However, unlike the mesorectum, which has well-defined anatomical boundaries, the mesopancreas lacks a distinct fascial envelope, making its identification and dissection more challenging [30]. This underscores the need for standardized surgical techniques and a thorough anatomical understanding to implement TMpE effectively. Advancements in imaging modalities and surgical approaches, such as the artery-first approach, have been developed to facilitate the identification and dissection of the mesopancreas, thereby enhancing the precision of TMpE [31,32].

### 4.5. Advances in Imaging

In recent years, advances in imaging have revolutionized the understanding of the mesopancreas, enhancing both diagnostic accuracy and surgical planning. High-resolution imaging modalities [32,33], such as magnetic resonance imaging (MRI) and computed tomography (CT), have emerged as indispensable tools for visualizing the mesopancreatic region [34,35]. These techniques provide detailed, multi-dimensional views of the pancreatic head, the surrounding vasculature, and the mesopancreas itself, allowing surgeons to map critical anatomical landmarks with precision. Contrast-enhanced CT, in particular, excels in delineating vascular structures and detecting tumor infiltration [35,36,37], whereas MRI is superior for assessing soft tissue involvement and identifying perineural or lymphatic spread. Its superior soft tissue contrast and multiplanar capabilities make it the preferred modality for evaluating PNS in head and neck cancers. MRI can depict perineural tumor spread with a sensitivity of 95%, although this sensitivity decreases to 63% when mapping the entire extent of the spread [38]. Advanced MRI techniques, such as diffusion-weighted imaging (DWI) and dynamic contrast-enhanced (DCE) MRI, further enhance diagnostic accuracy. DWI is sensitive to the Brownian motion of water molecules, allowing for differentiation between malignant and benign pancreatic lesions. Studies have shown that DWI can distinguish between pancreatic adenocarcinoma and mass-forming pancreatitis, aiding in accurate diagnosis and treatment planning [39]. Dynamic contrast-enhanced MRI provides additional information on tissue vascularity and perfusion, which is valuable in characterizing pancreatic tumors and assessing their response to therapy. The combination of DWI and DCE-MRI provides a comprehensive evaluation of pancreatic lesions, enhancing preoperative decision-making and reducing the likelihood of unexpected findings during surgery [40].

### 4.6. Technological Innovations

The advent of robotic and laparoscopic surgical techniques has brought a new dimension to the dissection of the mesopancreas. These minimally invasive approaches provide enhanced visualization, precision, and dexterity, enabling surgeons to navigate the intricate anatomy of the mesopancreatic region with greater confidence. Robotic platforms, in particular, have enabled more precise dissection around critical structures, such as the superior mesenteric artery (SMA), celiac plexus, and portal vein, thereby reducing the risk of intraoperative complications. A study published in the *Annals of Surgical Oncology* provided a comprehensive overview of mesopancreas dissection during robotic pancreatoduodenectomy (PD). The authors described three distinct levels of mesopancreas dissection tailored to tumor type and vascular anatomy, highlighting the advantages of robotic assistance in achieving precise and safe dissections [41]. Another study emphasized the development of the multiple scope transition (MST) method in robotic PD. This technique optimizes surgical views during various stages of the procedure, enhancing the efficiency and safety of mesopancreas dissection. The MST method underscores the benefits of robotic systems in providing superior visualization and maneuverability in complex anatomical regions [42]. Laparoscopic techniques, when combined with advanced imaging modalities such as intraoperative ultrasound (IOUS), enhance a surgeon’s ability to identify and excise tumor-infiltrated tissues accurately. IOUS provides real-time imaging during surgery, allowing for precise localization of tumors and assessment of their relationship to surrounding structures, which is particularly beneficial in pancreatic surgeries [43]. Preliminary studies have demonstrated that minimally invasive approaches, including robotic and laparoscopic surgeries, can achieve oncological outcomes comparable to those of open surgery. These techniques offer additional benefits, such as reduced recovery times and decreased postoperative pain. For instance, a study comparing robotic and laparoscopic distal pancreatectomy found that both approaches had similar perioperative and oncologic outcomes, with the robotic approach associated with a lower conversion rate to open surgery [44]. As these technologies continue to evolve, they hold the promise of setting new standards for the surgical management of pancreatic head malignancies, potentially improving patient outcomes and quality of life.

## 5. Conclusions

Based on our cadaveric dissections, we propose a plausible definition for the term “mesopancreas”. It refers to the structures that were originally part of the primordial mesenteries but disappeared through the process of coalescence, ultimately forming the Treitz fascia. In essence, the mesopancreas represents the functional remnants of a former mesentery.

These anatomical components—especially the neural and lymphatic pathways—may explain the high rate of local recurrence in pancreatic head cancer, making the mesopancreas a critical target in surgical resection. Despite its potential oncologic significance, the mesopancreas remains a technically challenging structure due to its depth and proximity to major vessels. A deeper understanding of this anatomical region will contribute to improved surgical precision and better oncological outcomes, while also fostering future interdisciplinary research.

## Data Availability

Data are contained within the article.
